# Structural and functional interactions between six-transmembrane μ-opioid receptors and β_2_-adrenoreceptors modulate opioid signaling

**DOI:** 10.1038/srep18198

**Published:** 2015-12-11

**Authors:** Alexander Samoshkin, Marino Convertino, Chi T. Viet, Jeffrey S. Wieskopf, Oleg Kambur, Jaclyn Marcovitz, Pinkal Patel, Laura S. Stone, Eija Kalso, Jeffrey S. Mogil, Brian L. Schmidt, William Maixner, Nikolay V. Dokholyan, Luda Diatchenko

**Affiliations:** 1 Alan Edwards Centre for Research on Pain, McGill University, Montreal, QC, H3A 0G1, Canada; 2 Department of Anesthesia, McGill University, Montreal, QC, H4A 3J1, Canada; 3 Department of Biochemistry and Biophysics, University of North Carolina, Chapel Hill, NC, 27599, USA; 4 Bluestone Center for Clinical Research, New York University, New York, NY, 10010, USA; 5 Department of Psychology, McGill University, Montreal, QC, H3A 1B1, Canada; 6 Department of Pharmacology, Faculty of Medicine, University of Helsinki, 00014 Helsinki, Finland; 7 Faculty of Dentistry, McGill University, Montreal, QC, H3A 1G1, Canada; 8 Department of Anesthesiology, Intensive Care and Pain Medicine, University of Helsinki and Helsinki University Hospital, 00290 Helsinki, Finland; 9 Center for Pain Research and Innovation, University of North Carolina, Chapel Hill, NC, 27599, USA

## Abstract

The primary molecular target for clinically used opioids is the μ-opioid receptor (MOR). Besides the major seven-transmembrane (7TM) receptors, the MOR gene codes for alternatively spliced six-transmembrane (6TM) isoforms, the biological and clinical significance of which remains unclear. Here, we show that the otherwise exclusively intracellular localized 6TM-MOR translocates to the plasma membrane upon coexpression with β_2_-adrenergic receptors (β_2_-ARs) through an interaction with the fifth and sixth helices of β_2_-AR. Coexpression of the two receptors in BE(2)-C neuroblastoma cells potentiates calcium responses to a 6TM-MOR ligand, and this calcium response is completely blocked by a selective β_2_-antagonist in BE(2)-C cells, and in trigeminal and dorsal root ganglia. Co-administration of 6TM-MOR and β_2_-AR ligands leads to substantial analgesic synergy and completely reverses opioid-induced hyperalgesia in rodent behavioral models. Together, our results provide evidence that the heterodimerization of 6TM-MOR with β_2_-AR underlies a molecular mechanism for 6TM cellular signaling, presenting a unique functional responses to opioids. This signaling pathway may contribute to the hyperalgesic effects of opioids that can be efficiently blocked by β_2_-AR antagonists, providing a new avenue for opioid therapy.

The μ-opioid receptor (MOR) is the main target for clinically used opioids, the most effective analgesic drugs for the treatment of moderate-to-severe pain. Long-term use of opioids, however, is compromised by adverse effects such as tolerance (reduced efficacy), physical dependence and opioid-induced hyperalgesia (OIH)^1^,^2^. The molecular mechanisms behind the development of these adverse effects are still poorly understood, but receptor desensitization, downregulation, methylation, decreased signal transduction and paradoxical stimulatory effects of MOR have been proposed.

The major isoform MOR1 is a seven-transmembrane (7TM) spanning G‐protein (Gαi/o)-coupled receptor (GPCR) encoded by the *OPRM1* gene. MOR1 activation, which is pivotal for the analgesic effect of opioids, leads to reduced excitability of neurons by inhibiting adenylyl cyclase and voltage-gated Ca^2+^ channels and by activating of K^+^ channels^3^. The *OPRM1* gene also codes for multiple alternatively spliced isoforms, including a new class of truncated isoforms, consisting of 6TM helices, lacking the first TM and the extracellular domain^4^,^5^.

At the cellular level, it has been shown that 6TM-MOR may play a particularly important role in altering MOR signaling^5^,^6^. In contrast to 7TM-MOR overexpressing cells, stimulation of cells expressing the 6TM-MOR variant MOR1K with morphine exhibit excitatory cellular responses exemplified by increased levels of intracellular Ca^2+^ as well as increased nitric oxide production and release^6^. However, the functional activity of 6TM-MOR has been questioned because it is not localized to the cell membrane when expressed alone, but is instead retained in the intracellular compartments following heterologous expression^6^,^7^. Furthermore, IBNtxA, which is a putative agonist of the 6TM splice isoforms, shows receptor binding to brain membranes from triple-knockout (KO) mice lacking 7TM opioid receptors but retain intact 6TM isoforms. However, IBNtxA fails to bind cells transfected with a 6TM isoform alone suggesting that truncated 6TM isoforms may require specific partners to become functional^7^.

Our search for the signaling partner for 6TM-MOR focused on the β_2_-AR for several reasons. First, β_2_-AR has been shown to chaperone other GPCRs; for example, physical and functional interactions between β_2_-AR and the CB1 cannabinoid receptor or the M71 olfactory receptor lead to trafficking of these GPCRs to the cell surface^8^,^9^. Second, the β-adrenergic pathway affects opioid signaling, and recent studies have revealed an important role of β_2_-ARs in opioid tolerance, physical dependence and opioid-induced hyperalgesia in rodent models^10^,^11^. In line with this, partially selective beta-blockers significantly decreases the postoperative consumption of opioids^12^. Finally, the fact that β_2_-ARs are ubiquitously expressed enables co-expression with 6TM-MOR in the majority of mammalian cells including neurons^13^,^14^,^15^.

To study the putative interactions between β_2_-AR and 6TM-MOR we combined computational analysis, biochemical and functional *in vitro* studies in BE(2)-C neuroblastoma transfected cells; *ex vivo* mice ganglion neurons and *in vivo* animal pain behavior tests. Our results provide evidence that the heterodimerization of 6TM-MOR with β_2_-AR underlies the molecular mechanisms for excitatory cellular signaling induced by opioids, which are blocked by β_2_-AR antagonists resulting in an enhancement of opioid analgesia and elimination of OIH.

## Results

### 6TM-MOR interacts with β_2_-AR upon heterologous expression

**Figure 1:**
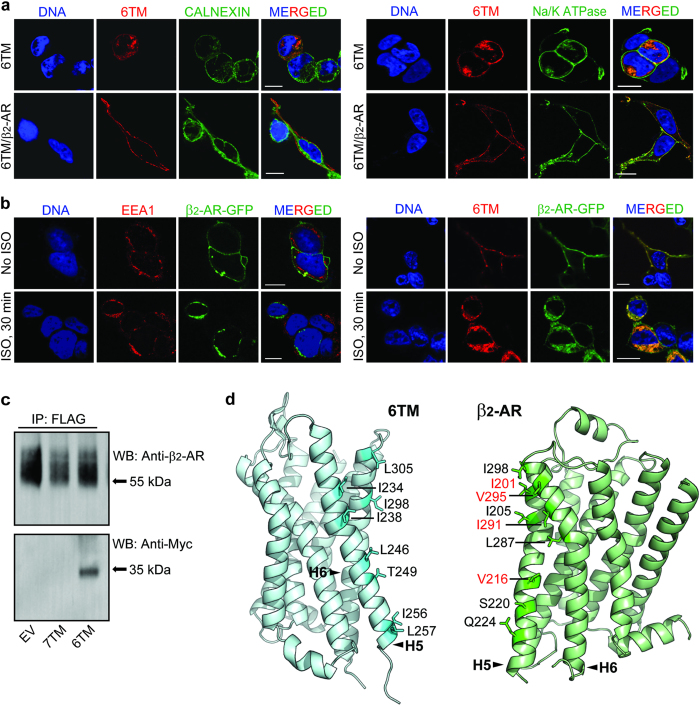
6TM-MOR interacts with β_2_-AR upon heterologous expression. (**a**) 6TM-MOR (anti-FLAG, red) was located mainly intracellularly (ER marker anti-calnexin, green, left panel) when expressed alone (top row of each panel), but translocated to the plasma membrane (anti-Na^+^/K^+^-ATPase, green, right panel) when co-expressed with β_2_-AR (bottom row of each panel) in HEK293 cells. (**b**) β_2_-AR and 6TM-MOR co-internalized in response to 10 μM isoproterenol stimulation (ISO). EEA1 was used as an early endosomes marker (red, left panel) and 6TM-MOR was visualized with anti-FLAG (red, right panel). For (a) and (b) DNA was counterstained with Hoechst 33342 (blue). Scale bar: 10 μm. (**c**) Myc-tagged 6TM-MOR, but not 7TM-MOR, was immunoprecipitated with β_2_-AR from HEK293 cell lysates stably expressing FLAG-tagged β_2_-ARs. IP: precipitating antibody; WB: probing antibody; EV: empty vector. (**d**) Residues mediating the interaction between 6TM-MOR and β_2_-AR were identified along the putative surface of heterodimerization between helices H5 and H6 of 6TM-MOR and β_2_-AR. Residues predicted to mediate the interaction are indicated. When 6TM-MOR was co-expressed with the β_2_-AR mutant consisting of four alanine substitutions of the residues indicated in red, 6TM-MOR localized to intracellular compartments (Supplementary Fig. S1).

Because 6TM-MOR is not localized to the cell membrane when over-expressed in heterologous cellular systems^6^,^7^, we first tested if β_2_-AR is able to chaperone 6TM-MOR to the cell surface. We expressed 6TM-MOR tagged with a FLAG epitope alone or in combination with each AR (untagged) subtypes (β_1_-, β_2_- and β_3_-ARs) in HEK293 cells. We then screened the cells, using confocal microscopy, for 6TM-MOR localization. We observed that, when expressed alone, 6TM-MOR is primarily present in the intracellular compartment and co-localized with the endoplasmic reticulum (ER), and only a small fraction is found in the plasma membrane (Fig. 1a, upper rows). When co-expressed with β_2_-AR, 6TM-MOR translocates from ER compartments to the plasma membrane (Fig. 1a, lower rows). In contrast, upon co-expression with β_1_- or β_3_-AR, 6TM-MOR is retained within its intracellular compartments, suggesting that β_2_ is the only subtype of β-ARs responsible for 6TM-MOR translocation. Co-expression of 6TM-MOR with 7TM-MOR, delta-, kappa- or ORL1 receptors also does not lead to 6TM-MOR translocation to the cell surface membrane (data not shown).

To further confirm the physical association between 6TM-MOR and β_2_-AR, we determined whether exposure to the non-selective β-AR agonist isoproterenol (ISO) causes internalization of the two receptors. Agonist stimulation of β_2_-ARs leads to rapid desensitization and trafficking of the receptors to lysosomes^16^,^17^,^18^, which we visualized and confirmed with the early endosomes marker (EEA1; Fig. 1b, left panel). We observed that stimulation of 6TM-MOR-FLAG and β_2_-AR-GFP co-transfected cells with 10 μM ISO for 30 min results in the internalization of both 6TM-MOR and β_2_-AR (Fig. 1b, right panel).

To show that 6TM-MOR translocation to the cell surface is due to a direct biochemical interaction with β_2_-AR, we expressed 7TM- or 6TM-MOR (Myc-tagged) in HEK293 cells stably expressing FLAG-tagged β_2_-AR. Cell lysates were subjected to immunoprecipitation with anti-FLAG antibodies and the pulled-down proteins were then detected by Western blotting. The presence of β_2_-AR protein was confirmed in all three immunoprecipitates; strikingly, Myc immunostaining was observed almost exclusively in the sample obtained from Myc-6TM-MOR transfected cells (~35kDa), suggesting that 6TM-MOR, but not 7TM-MOR, is the primary partner for interacting with β_2_-AR (Fig. 1c).

In order to provide a detailed description of the interactions between 6TM-MOR and β_2_-AR on the structural level, we modeled the structure of the 6TM-MOR/β_2_-AR heterodimer by performing protein-protein docking calculations^19^ followed by short discrete molecular dynamics (DMD) simulations^20^,^21^. The putative surface supporting heterodimerization was predicted to occur between helices H5 and H6 of 6TM-MOR and β_2_-AR, which is analogous to the surfaces mediating MOR homodimerization^22^. Based on this *in silico* structural model of the 6TM-MOR/β_2_-AR heterodimer, we identified a series of amino acids in the two GPCRs that mediate the formation of the heterodimer (Fig. 1d). Then, we performed site-directed mutagenesis with the goal of disrupting the van der Waals interactions between pairs of residues at the heterodimerization surface of monomeric 6TM-MOR and β_2_-AR. When 6TM-MOR was co-expressed with the β_2_-AR mutant featuring an alanine substitution of the I201 residue, 6TM-MOR translocation to the plasma membrane was diminished (Supplementary Table 1). Furthermore, after 6TM-MOR co-expression with the β_2_-AR mutant with alanine substitutions of four residues predicted to mediate the formation of the 6TM-MOR/β_2_-AR heterodimer (i.e., I201A, V216A, V295A, I291A; Fig. 1d), 6TM-MOR translocation to the plasma membrane was completely abrogated and 6TM-MOR remained confined to the intracellular compartment (Supplementary Fig. S1 and Table 1). After 6TM-MOR co-expression with the β_2_-AR mutants carrying alanine substitutions of five other residues located on the putative surface of heterodimerization between helices H5 and H6 (Fig. 1d and Supplementary Table 1), or residues not facing the surface of heterodimerization, 6TM-MOR relocation to the plasma membrane was equivalent to those of wild type β_2_-AR (Supplementary Fig. S1, Fig. 1a, bottom row of each panel).

These findings imply that predicted mutations of residues located on helices 5 and 6 of β_2_-AR disrupt the 6TM-MOR/β_2_-AR heterodimer, and prevent the trafficking of 6TM to the cell surface. On the other hand, none of the mutations in helices 5 and 6 of 6TM-MOR (Fig. 1c and Supplementary Table 1) resulted in the retention of monomeric 6TM-MOR in the intracellular compartments, which is likely due to one of the following scenarios. First, it is possible that the number of mutations introduced in 6TM-MOR was not sufficient to promote the complete disruption of the 6TM-MOR/β_2_-AR heterodimer. Second, the mutations may cause a conformational rearrangement that did not disrupt the heterodimerization surface in 6TM-MOR. Third, it is possible that 6TM-MOR heterodimerized with β_2_-AR by exploiting a different surface interaction. Finally, we cannot exclude the possibility that a third, as yet unidentified protein mediates the physical interaction between β_2_-AR and 6TM-MOR. Nevertheless, our computational and experimental findings suggest that β_2_-AR can effectively heterodimerize with 6TM-MOR, serve as molecular chaperone, and ultimately, promote the trafficking of this MOR isoform from the intracellular compartments to the plasma membrane.

### β_2_-AR contributes to 6TM-dependent Ca^2+^ response induced by 6TM-MOR ligand in a heterologous expression system and mouse ganglia neurons

**Figure 2:**
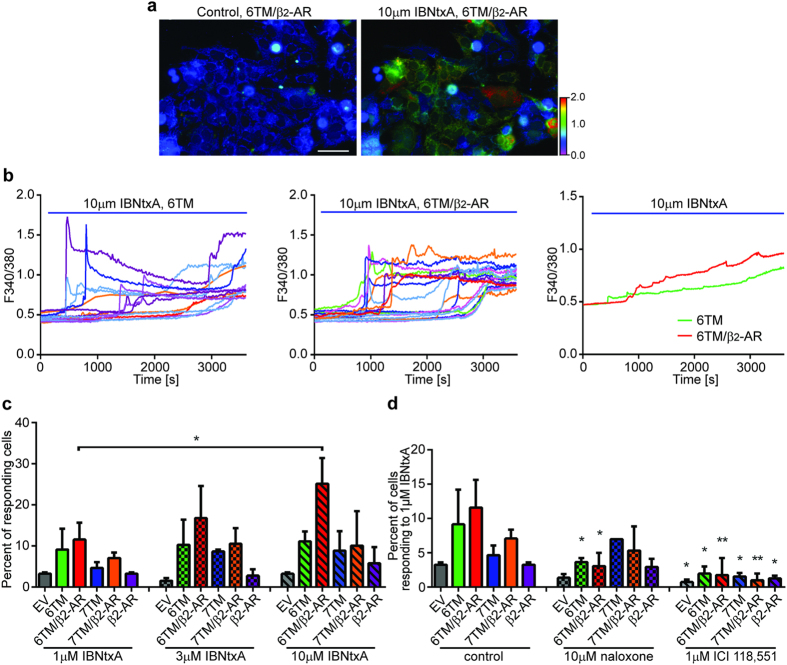
β_2_-AR contributes to the 6TM-dependent Ca^2+^ response upon treatment by 6TM-MOR ligand IBNtxA. (**a**) Representative images of BE(2)-C cells co-transfected with 6TM and β_2_-AR before (left; perfused with PBS) and after IBNtxA treatment (right; perfused with 10 μM IBNtxA). A range of the Ca^2+^ responses were imaged using Fura-2 ratiometric dye. Color scale from green to red shows the F340/380 ratio increase. Scale bar: 100 μm. (**b**) Ca^2+^ responses of individual cells, presented as increases in F340/380 for cells transfected with 6TM-MOR (left) or 6TM-MOR/β_2_-AR (middle). 10 μM IBNtxA was perfused between 60–3600 sec (blue bar). Averaged tracings of Ca^2+^ -responding cells are shown on right. **(c**) The percentage of Ca^2+^ -responding cells upon increasing concentrations of IBNtxA. *p < 0.05, one-way ANOVA and Sidak’s multiple comparison test. Perfusion conditions: 1 min PBS; 59 min 1, 3 or 10 μM IBNtxA. (**d**) The percentage of Ca^2+^ -responding cells treated with 1 μM IBNtxA was reduced either by non-selective opioid antagonist naloxone or by the selective β_2_-AR antagonist ICI 118,551. Perfusion conditions: 1 min PBS-3 min 10 μM naloxone or 1 μM ICI 118,551;-56 min 10 μM naloxone/1 μM IBNtxA or 1 μM ICI 118,551/1 μM IBNtxA respectively. *p < 0.05, **p < 0.01, Student’s t-test vs. control.

To elucidate the functional role of 6TM MOR/β_2_-AR heteromerization *in vitro* we used the recently developed 6TM-MOR ligand, IBNtxA^7^. We measured IBNtxA-dependent dynamics on intracellular Ca^2+^ levels because morphine activation of the 6TM-MOR receptor leads to an increase in intracellular Ca^2+^^6^. In these studies, we over-expressed either 6TM- or 7TM-MOR alone, or in conjunction with β_2_-AR, in neuroblastoma BE(2)-C cells and then quantified IBNtxA-dependent Ca^2+^ responses with real-time imaging following ligand application (Fig. 2a,b). BE(2)-C cells co-expressing 6TM-MOR and β_2_-AR showed a robust Ca^2+^ response that was significantly higher (25.2% of BE(2)-C cells, 10 μM IBNtxA) than in cells co-expressing 7TM-MOR and β_2_-AR (10.1% of BE(2)-C cells), or cells expressing only 6TM-MOR, 7TM-MOR, β_2_-AR, or EV (11.1%, 8.8%, 5.8% or 3.3% of BE(2)-C cells, respectively; Fig. 2c). Both the percentage of responding cells (Fig. 2c) and the amplitude of Ca^2+^ response (Fig. 2b) were higher in cells that co-expressed 6TM-MOR and β_2_-AR. Furthermore, the IBNtxA-induced Ca^2+^ response in 6TM-MOR/β_2_-AR expressing cells was dose-dependent: 11.6%, 16.8%, and 25.1% of co-transfected BE(2)-C cells responded to 1, 3 and 10 μM IBNtxA, respectively (Fig. 2c). We next determined the effect of pharmacologically antagonizing either 6TM-MOR or β_2_-AR on IBNtxA-induced Ca^2+^ responses, using the non-selective opioid receptor antagonist, naloxone, or the selective β_2_-AR antagonist, ICI 118,551 (Fig. 2d). Pre-treatment with 10 μM naloxone significantly inhibited IBNtxA-induced Ca^2+^ responses in cells that either over-expressed 6TM alone or 6TM/β_2_-AR (3.9% and 3.1% of BE(2)-C cells, respectively). Naloxone pre-treatment did not further inhibit IBNtxA response in cells that over-expressed either 7TM-MOR or 7TM-MOR/β_2_-AR, suggesting that, in the Ca^2+^ assay, IBNtxA acted specifically on 6TM but not on 7TM MOR. Pre-treatment with ICI 118,551 resulted in a significant inhibition of IBNtxA-induced Ca^2+^ responses in all treatment groups (Fig. 2d). These results reveal the central role of β_2_-AR as a mediator of 6TM-MOR-dependent increase in intracellular Ca^2+^ levels, which is consistent with excitatory, rather than inhibitory, cellular effects of opioids.

**Figure 3:**
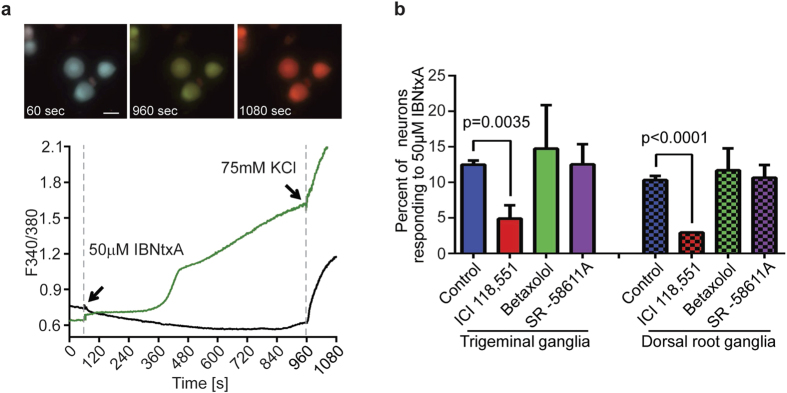
Ca^2+^ response to IBNtxA in mouse ganglia neurons is dependent on β_2_-adrenergic receptor activity. (**a**) Representative time-lapse photos of dissociated mouse dorsal root ganglia at baseline (60 sec), responding to 50 μM IBNtxA (960 sec), and responding to 75 mM KCl (1080 sec). Scale bar = 20 μm. The graph represents 340 nm/380 nm ratio over the experimental time course. The neurons display a stable baseline during the first minute while perfused with HBSS buffer. 50 μM IBNtxA and 75 mM KCl are added at the indicated times. Only neurons with a positive KCl response are included in the analysis. (**b**) The bar graph shows the proportion of neurons with a positive response to 50 μM IBNtxA in the presence of the indicated selective antagonist. For both trigeminal neurons and dorsal root neurons, pre-treatment (3 min) with the selective β_2_-adrenergic receptor antagonist, 0.3 μM ICI 118,551, significantly inhibits the Ca^2+^ response to IBNtxA. Pre-treatment with the β_1_-AR selective antagonist, 0.3 μM betaxolol, and the β_3_-AR selective antagonist, 0.1 μM SR-58611A, does not significantly affect the Ca^2+^ response to IBNtxA (Student’s t-test, p-values as indicated).

Sensory dorsal root ganglia (DRG) and trigeminal ganglia (TG) neurons encode nociceptive signals that give rise to the perception of pain, and are modulated by MOR activation via effects on voltage-gated Ca^2+^ channels. Thus, neurons within these ganglia are important endogenous sites of action of the effects of opioids on nociceptive transmission^23^. Endogenous levels of expression of MORs and β_2_-AR in neurons isolated from naïve mice are low which brings challenges for functional assays in terms of obtaining reliable responses^15^. We examined the effects of IBNtxA administration on Ca^2+^ responses in *ex vivo* neurons and determined if the evoked responses were dependent on β_2_-ARs (Fig. 3a). We harvested and dissociated mouse trigeminal and dorsal root ganglia, and observed that 12.5% of TG and 10.3% of DRG neurons are activated by 50 μM IBNtxA (a lower concentration of 10 μM IBNtxA produced inconsistent activation). Morphologically, the activated neurons were medium sized 15–20 μm in diameter. When pretreated with a β_1_-, β_2_-, or β_3_-AR selective antagonists betaxolol, ICI 118,551 or SR-58611A, respectively, IBNtxA-dependent Ca^2+^ responses observed in both TG and DRG neurons were blocked only by the selective β_2_-AR antagonist ICI 118,551 (5% of TG and 3% of DRG neurons, respectively; Fig. 3b). These results indicate that *ex vivo* isolated neurons respond to IBNtxA treatment with increasing intracellular Ca^2+^ concentration, and this response is dependent on β_2_-ARs, but not β_1_-ARs or β_3_-ARs. Although we do not have a selective 6TM-MOR agonist available as a research tool, these results are consistent with 6TM-MOR/β_2_-AR heterodimer signaling observed in the heterologous expression system.

### β_2_-AR antagonist produces synergy in acute analgesic effects and reduces OIH after chronic administration of opioid ligands

**Figure 4:**
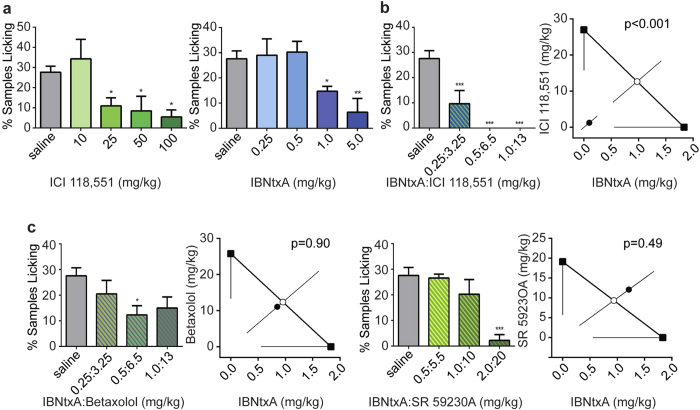
Co-administration of IBNtxA and β_2_-AR antagonist produces robust synergy in analgesic efficacy. (**a**) β_2_-AR antagonist ICI 118,551 and 6TM-MOR ligand IBNtxA produce dose-dependent analgesia. Graphs show late phase (10–60 min post-injection) nocifensive behaviors after 5% formalin injection into the plantar hind paw; bars represent mean percentage of 10-sec mice (1 mouse/min) featuring licking behavior directed toward the injected paw (% mice licking). Error bars represent SEM. Left graph shows that the 25, 50 and 100 mg/kg doses of ICI 118,551 significantly inhibit pain behavior compared to saline. Right graph shows that the 1 and 5 mg/kg doses of IBNtxA differ significantly from saline. (**b**) Co-administration of ICI 118,551 and IBNtxA produces synergistic analgesia. Left graph shows the dose response curve of the cocktail tested), and the right graph shows the isobolographic analysis, confirming the synergistic relationship of these two drugs (p < 0.001). **(c)** Neither β_1_-AR antagonist betaxolol nor β_3_-AR antagonist SR 59230A show a synergistic interaction with IBNtxA. Left graph shows the dose response curve of the cocktail tested, and the right graph shows the isobolographic analysis, showing no synergistic relationship of IBNtxA and either betaxolol or SR 59230A (p = 0.90 and 0.49, respectively). *p < 0.05, **p < 0.01, ***p < 0.001.

To demonstrate the potential biological and clinical significance of 6TM-MOR/β_2_-AR heterodimer signaling, we performed *in vivo* animal studies to investigate the synergistic interaction between opioid (morphine and IBNtxA) and β_2_-AR (ICI 118, 551) ligands using the mouse (5% plantar) formalin test. Because the activation of the 6TM-MOR/β_2_-AR heterodimer seemed to produce cellular excitatory signaling, we proposed that 6TM-MOR/β_2_-AR heterodimer signaling would contribute to a reduction in the analgesic efficacy of MOR agonists. We expected, therefore, that the co-administration of 6TM-MOR agonists and β_2_-AR antagonists would disrupt the excitatory dimer signaling, and produce analgesic synergy. To determine whether the interaction between IBNtxA and β_2_-AR antagonist ICI 118,551 is indeed synergistic, we conducted an isobolographic analysis. As expected, a dose-dependent analgesic response following the administration of either IBNtxA or ICI 118,551 alone was observed in both early (Supplementary Fig. S2) and late phase of formalin test (Fig. 4). Since the late phase of the formalin test showed much more robust response and it is thought to have more clinical relevance^24^, we then focused on the late phase responses only. Co-administration of IBNtxA and ICI 118,551 induced an analgesic response much greater than the expected additive effect of the two drugs alone (p = 0.0003, Fig. 4b). Neither the β_1_-AR antagonist betaxolol nor the β_3_-AR antagonist SR 59230A showed a similar synergistic interaction with IBNtxA (p = 0.9 and 0.5, respectively, Fig. 4c and Supplementary Fig. S3a). These results are consistent with the observed cellular responses dependent on the 6TM-MOR/β_2_-AR heterodimer, which is specific for β_2_-ARs (Fig. 2). A similar synergistic effect, although of smaller magnitude, was observed between morphine and ICI 118,551, but not between morphine and betaxolol or SR 59230A (Supplementary Fig. S3b). Although we cannot exclude that the observed analgesic response originates from the interactive effect of these drugs on opioid and adrenergic signaling pathways independently that do not involve a physical interaction between 6TM-MOR and β_2_-AR, these results are consistent with the concept of 6TM-MOR/β_2_-AR heterodimer formation, and with the hyperalgesic signaling of the heterodimer. Furthermore, the observation that the magnitude of a synergistic effect of IBNtxA and ICI 118,551 is much more robust than that of morphine is consistent with the relative binding affinities of these ligands to the 6TM-MOR isoform^7^.

**Figure 5:**
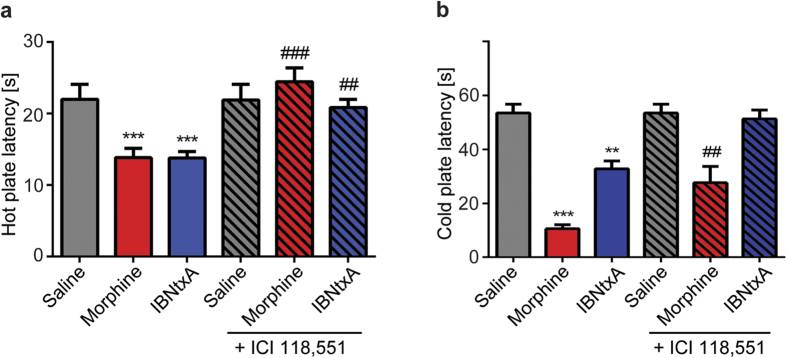
β_2_-AR antagonist ICI 118,551 blocks OIH after chronic morphine or IBNtxA administration. C57BL/6J mice received 4 days of morphine (20 mg/kg days 1–3, 40mg/kg day 4, s.c., twice a day, n = 15), or IBNtxA (2 mg/kg days1–3, 4 mg/kg day 4, s.c., twice a day, n = 15) treatment to induce OIH or vehicle (saline, s.c., n = 12). Chronic morphine or IBNtxA administration produces OIH assessed in thermal tests. (**a,b**) β_2_-AR antagonist ICI 118,551 (3.25mg/kg, s.c.) restores nociceptive responses to thermal stimuli back to baseline levels. Nociceptive latencies were not affected in vehicle treated animals (n = 8). (**a**) Thermal hyperalgesia was assessed by hot plate test and (**b**) cold allodynia by cold plate test. Error bars represent SEM. **p < 0.01, ***p < 0.001 vs. saline; ^**##**^p < 0.01, ^**###**^p < 0.001 vs. morphine or IBNtxA baseline respectively (two-way ANOVA combined with Tukey’s multiple comparison test).

Since excitatory MOR signaling has been linked to OIH we decided to study this phenomenon in a mouse model based on repeated opioid administration for 4 days^10^,^11^. On the fifth day of experiment, mice treated with morphine or IBNtxA were tested for thermal and mechanical sensitivity (Fig. 5; Supplementary Fig. S4). Mice displayed significant increases in sensitivity to mechanical stimuli (Supplementary Fig. S4; p < 0.001) and shorter latencies on hot and cold plates compared to the vehicle groups (Fig. 5; p < 0.001, vs. saline).

After verifying the development of OIH in mice we proceeded to study if treatment with ICI 118,551 could reverse the thermal sensitivity. OIH baseline was measured first, followed by a single injection of ICI 118,551 (3.25mg/kg, s.c.). At this dose ICI 118,551 efficiently blocks β_2_-ARs, but lacks independent analgesic effects as shown in acute experiments (Supplementary Fig. S3). We observed a complete reversal of thermal hyperalgesia in the hot plate test by ICI 118,551 (Fig. 5a; p < 0.001, p < 0.01, vs. morphine or IBNtxA baseline respectively). β_2_-AR antagonist did not alter the latencies in the vehicle group. Similar effect was observed in the cold plate test for IBNtxA whereas for morphine the reversal was partial (Fig. 5b). The vehicle injected animals did not develop OIH and we did not observe any differences in nociceptive responses in the hot and cold tests after single injection of ICI 118,551 in comparison with the vehicle group before injection (Fig. 5). These results further reinforce our hypothesis that the stimulation of 6TM-MOR/β_2_-AR heterodimers drives intracellular signaling that produced the hyperalgesic effects of 6TM opioid agonists and suggest that disrupting 6TM-MOR/β_2_-AR heterodimers with β_2_-AR antagonists holds potential to reduce certain adverse effects of opioids.

## Discussion

In the presented set of studies we provide evidence that the 6TM-MOR isoform heterodimerizes with β_2_-AR, and that this dimerization underlies a molecular mechanism for 6TM cellular signaling that mediates the excitatory and hyperalgesic effects of opioids. The co-expression and dimerization of these two receptors results in the translocation of the 6TM-MOR isoform from an intracellular compartment to the plasma membrane. The outcomes of our *ex vivo* studies suggest that this complex is expressed at low levels on a few sensory neurons. Stimulation of this complex by IBNtxA activates the signaling of 6TM-MOR/β_2_-AR heterodimers, which can be blocked by the selective β_2_-AR antagonist ICI 118,551. Finally, co-administration of 6TM-MOR and β_2_-AR ligands leads to substantial analgesic synergy and completely reverses OIH in animal models of pain.

The results have broad basic molecular and cellular implications. Although there has been a recent recognition that receptor heterodimerization adds another important dimension to the diversity of cellular responses controlled by GPCRs, the scope of these effects is just starting to emerge^25^,^26^. A few examples of GPCR heterodimers with functional properties are: μ-opioid and α2A-adrenergic receptors^27^; β_2_-ARs in complex with olfactory receptors^8^; GABA_B_ receptor subtypes^28^; ghrelin receptors^29^; neurotensin NTS_1_ and NTS_2_ receptors^30^. It has been shown that activation of either μ-opioid or α2A receptor leads to increased G-protein coupling, whereas simultaneous activation of both receptors leads to an attenuated response in HEK293 cells and in primary spinal cord neurons^27^. Coexpression of β_2_-ARs is required for activation of wild-type M71 olfactory receptors^8^. The GABA_B_ receptor (GABA_B_R1) is not effectively coupled to expected signaling pathways until co-expressed with GABA_B_R2, which allows GABA_B_1R to escape from the ER^31^,^32^. Furthermore, a functionally inactive truncated GHS-R1b can generate a novel pharmacology by heterodimerization with another GPCR NTS_1_ creating a receptor complex responsive to neuromedin U^30^. Our data add another facet to current knowledge of GPCR heteromerization and demonstrate how receptor heteromerization may change the direction of opioid cellular signaling, from inhibitory to excitatory. Furthermore, because a specific isoform of the MOR receptor heterodimerizes with β_2_-AR unmasks a novel cellular signaling pathway, further investigation of the functional importance of 6TM isoforms in modifying GPCR signaling is warranted.

Our findings may also have considerable clinical significance. Opioids remain the most commonly used drugs for treatment of moderate-to-severe pain. Opioid analgesia is largely facilitated by the inhibition of synaptic transmission, and MOR-mediated inhibition of voltage-gated Ca^2+^ channels on central presynaptic terminals of primary afferent nociceptors is thought to be one of the primary mechanisms mediating analgesia at the spinal level^23^,^33^. However, a concentration- and time-dependent effect of opioids on excitatory cellular responses has also been reported, and is associated with reduced analgesic efficacy, enhanced tolerance, OIH and dependence^34^. The molecular mechanism of these opioid-dependent cellular excitatory effects remains poorly understood.

Current understanding of 6TM-MOR stimulation profile is favorable towards traditional inhibitory opioid mediated effects, and IBNtxA, like morphine, produces analgesia following acute administration by stimulating 6TM-MORs^7^. However, our cellular results suggest that 6TM-MOR/β_2_-AR heterodimerization represents one of the molecular mechanisms underlying the excitatory cellular effects and produce enhanced nociception. Consistent with the cellular results, our animal data demonstrate synergistic analgesic interactions between 6TM-MORs agonist and β_2_-ARs antagonist. Furthermore, our findings also provide evidence that 6TM-MOR/β_2_-AR complex is signaling *in vivo* in response to repeated exposures to IBNtxA and contributes significantly to opioid mediated OIH as has been shown for morphine^10^,^11^. The observation that ICI 118,551 is able to effectively block heat and cold-evoked hyperalgesia suggests that at least a subset of sensory neurons that mediate hyperalgesia express 6TM-MOR/β_2_-AR heterodimers. Whether the sensitization of primary afferent neurons by various analgesics produces an increase in the expression of 6TM-MOR/β_2_-AR dimers in primary sensory neurons is an interesting question that requires further investigation. However, it is clear that prolonged use of opioids up-regulates mRNA expression of the majority of MOR isoforms; for example in the brainstem, all 6TM variants were reported to be significantly increased (up to 100 folds), suggesting a greater effect upon the exon 11 promoter than upon the exon 1 promoter in this conditions^35^. And in some tissues, like in the prefrontal cortex, 7TM variants appear to correspond to only a small fraction of full-length mRNA, implicating an important role of 6TM variants^35^ in modulating prefrontal cortical function.

The analgesic properties of IBNtxA in mouse models^36^ suggest that monomeric 6TM-MORs localized in the intracellular compartment or those heterodimerized with other GPCRs are responsible for the analgesia seen after the acute administration of IBNtxA and that more prolonged exposure to IBNtxA, and perhaps other opioids, results in an increase in the expression of the 6TM-MOR/β_2_-AR complex that functions to antagonize the analgesic properties mediated by 6TM- and 7TM-MORs. Consistent with this model, the reported improved adverse events profile of IBNtxA is related to its inhibitory effects associated with acute administration. The initial characterization of acutely administered IBNtxA concluded that respiratory depression and physical dependence are negligible and associated with limited inhibition of gastrointestinal transit was seen by IBNtxA^37^. It is also important to note that there is no current evidence suggesting adverse effects of ICI 118,551 on cardiovascular function (blood pressure, heart rate, vascular resistance)^38^,^39^,^40^.

Thus, in accordance with our conceptual model, the hyperalgesic effects of an opioid agonist derived from 6TM-MOR/β_2_-AR heterodimer can be efficiently blocked by a β_2_-AR antagonist and suggests that in the clinical setting, the simple co-administration of a β_2_-AR antagonist with opioid drugs may enhance the analgesic efficacy of opioids and diminish unwanted adverse effects, such as OIH.

Finally, our findings have profound implications on understanding the molecular processes that contribute to stress and opioid induced hyperalgesia, which have been linked to β_2_-AR activation. Circulating epinephrine, which is derived from the adrenal medulla, is the primary endogenous agonist for β_2_-ARs and mediates the effects of life relevant stressors on pain sensitivity^41^,^42^,^43^ and contributes to clinical pain states^44^,^45^,^46^,^47^. Similarly, the activation of β_2_-ARs plays a critical role in the production of opioid-induced tolerance and hyperalgesia, which are common clinical problems^10^,^11^,^48^,^49^. As such, it remains an interesting and open question as to the role that 6TM-MOR/β_2_-AR heterodimers play in these clinical states and in other clinical pain states that may result in the expression of 6TM-MOR/β_2_-AR (e.g. injury evoked hyperalgesia and pain).

In summary, our results contribute to the emerging effort to identify new therapeutic targets that selectively modulate inhibitory opioid receptor signaling while reducing the adverse effects associated with opiate use.

## Methods

### Cell culture

HEK293T and human neuroblastoma BE(2)-C cells (ATCC, Manassas, VA, USA) were maintained and transfected with Lipofectamine 2000 reagent (Life Technologies) according to the manufacturer’s instructions as described previously^6^. The HEK293 cell line with stable expression of FLAG-β_2_-AR was a gift from Dr. R. Lefkowitz (Duke University Medical Center, Durham, NC, USA)^50^. Mouse trigeminal and dorsal root ganglia neurons were harvested and cultured as previously described^51^. Trigeminal ganglia were removed, transferred into Hank’s Balanced Salt Solution (HBSS) and enzyme-digested by incubation with papain, collagenase type II (Worthington Biochemical Corp.) and dispase type II (Sigma-Aldrich). Dissociated neurons were plated on glass coverslips coated with poly-d-lysine and laminin and maintained at 37 °C at 5% CO_2_/95% air in F12 media (Life Technologies) with 5% FBS.

### Plasmid constructs

Human pIRES-EGFP-7TM and -6TM-MOR (no tag) expression constructs, and pIRES-EGFP-Myc-7TM and -6TM constructs were described previously^6^. The expression vector for human 6TM-MOR contains the sequence for the receptor coded by most human alternatively spliced *OPRM1* 6TM-MOR mRNAs: OPRM1K1, OPRM1K2, OPRM1-JL, OPRM1G1, but not MOR-3 and OPRM1-W (have alternative C-terminus) or OPRM1G2 (has additional N-terminus peptide). N-terminus FLAG-tagged (DYKDDDDK) 6TM WT and series of 6TM mutants (Supplementary Table 1) 1) I234A; 2) I234A, I256A, L305A, I298A; 3) L231A, I234A, I238A; 4) L231A, I234A, I238A, M243A, L257A; 5) I234A, I256A, L305A, I298A, L246A, V306A and 6) V126A, L116A, L121A, L129A, I322A were generated by site-directed mutagenesis (Integrated Technology Enterprise Inc., Atlanta, GA, USA) and inserted into pUNIV-SIG-FLAG expression vector (Addgene, Cambridge, MA, USA) using EcoRV/StuI restriction sites. pOPRSVI/MCS-β_2_-AR WT construct was provided by V. Setola (University of North Carolina at Chapel Hill, NC, USA). β_2_-AR mutants 1) I201A; 2) I201A,V216A, I291A, V295A; 3) O205A, S220A, Q224A, I298A, L297A; 4) V117I; 5) F298Y; 6) F290T; 7) V117I, F298Y, F290T were generated by site-directed mutagenesis (Integrated Technology Enterprise Inc., Atlanta, GA, USA) and cloned into the pOPRSVI/MCS vector. All constructs were verified by double-stranded DNA sequencing.

### Immunofluorescence microscopy

HEK293T cells were plated onto glass coverslips and transfected with plasmids encoding FLAG-tagged 6TM-MOR alone or with pOPRSVI/MCS-β_2_-AR as described above. 48 h after transfection, cells were fixed in -20^°^C methanol for 10 min, permeabilized with 0.25% Triton X-100 and blocked with 5% BSA for 40 min followed by immunostaining (primary antibody, overnight at 4 °C). Primary anti-FLAG (DYKDDDDK) antibody (Sigma-Aldrich) was used at a 1:1,000 dilution, anti-Calnexin (Abcam) and anti-Na/K ATPase α1 (Santa Cruz Biotechnology) antibodies were diluted 1:300, and anti-β_2_-AR 1:500 (Thermo Fisher Scientific). After washing with TBS, coverslips were incubated with appropriate secondary antibodies conjugated to Alexa Fluor 488 or 594 (Life Technologies) together with Hoechst 33342 (Sigma-Aldrich) for 1 h at room temperature. Samples were imaged using a Zeiss LSM 710 confocal microscope and images were collected and analyzed with ZEN Black software (Carl Zeiss).

### Immunoprecipitation (IP)

HEK293 cells stably expressing FLAG-tagged β_2_-ARs^50^ were transfected with Myc-tagged pIRES-EGFP-Myc-7TM or pIRES-EGFP-Myc-6TM constructs, or pIRES-EGFP-Myc empty vector (EV), and harvested at 48 h post-transfection. Pelleted cells were resuspended by passing through 22 g needle in ice-cold Pierce IP lysis buffer (Thermo Fisher Scientific). All further procedures were performed at +4 °C. After 15 min extraction, insoluble materials were removed by centrifugation (15,000 × g, 15 min) and the supernatant was used for IP. 400 μg of cellular extract was incubated with anti-FLAG M2 magnetic beads (Sigma-Aldrich) overnight. Beads were collected and washed 3 times with IP buffer and bound proteins were eluted with 3X FLAG peptide (Sigma-Aldrich), according to manufacturer’s instructions and then boiled in SDS-PAGE buffer. SDS-PAGE analysis and Western blotting were performed using the primary anti-β_2_-AR (1:2,000; Santa Cruz Biotechnology) and anti-Myc (EQKLISEEDL) antibodies (1:1,500; EMD Millipore).

### Generation of 6TM-MOR structural model

We generated the structural models of 6TM-MOR by applying minor changes to the crystallographic coordinates of the MOR chimera (PDB ID: 4DKL)^22^,^52^. We removed the coordinates of the co-crystallized morphinan antagonist β-funaltrexamine (β-FNA), of the first trans-membrane helix (i.e., from M65 to K98) and the T4 lysozyme. Additionally, we reconstructed the amino acid sequence of the i3 loop (i.e., from M264 to K269) and modeled its conformation using discrete molecular dynamics (DMD)^20^,^21^. A united atom representation was used to model protein structure, and the Lazaridis-Karplus implicit solvation model accounted for the solvation energy^53^. Temperature was controlled with the Andersen thermostat^54^. We ran a short DMD simulation (100,000 steps, i.e., ~5 ns) at the temperature of 0.5 kcal/mol k_B_ (~300 K). With the exception of atoms belonging to the i3 loop, all atomic coordinates from the crystal structure were harmonically constrained. We generated a total number of 1,000 loop conformations and, performed a cluster analysis based on the root mean squared distance (RMSD) of backbone-Cα atoms (applied cutoff of 2.5 Å). Finally, we retrieved the centroid of the most populated cluster as the representative conformation of the i3 loop in the 6TM-MOR structural model. We then assessed the quality of our generated model using Gaia^55^, (http://troll.med.unc.edu/chiron/login.php), which compares their intrinsic structural properties to high-resolution crystal structures. No critical issues were found in our 6TM-MOR model.

### Generation of the structural model of 6TM/β_2_-AR heterodimer

Starting from the X-ray structure of β_2_-AR at 2.8 Å resolution (PDB ID: 3NY80)^52^,^56^, and from the coordinates of 6TM-MOR derived as described above we generated the structural model of 6TM-MOR/β_2_-AR heterodimer by performing protein-protein docking calculations using the online server ZDOCK^19^. Guided by the structural model of the 7TM homodimer proposed by Manglik *et al.*, we restricted the docking exploration to protein surfaces corresponding to the transmembrane helices 5 and 6 in both 6TM-MOR and β_2_-AR. We retrieved the docking top-ranked solution of the 6TM-MOR/β_2_-AR heterodimer, and then optimized its structure using DMD^20^,^21^. In DMD atomic interactions are approximated by square-well potentials. The simulation engine solves a series of two-body collisions, in which colliding atoms’ velocities change instantaneously according to the conservation laws of energy, momentum, and angular momentum. A united atom representation was used to model protein structure, and the Lazaridis-Karplus implicit solvation model^57^ was adopted to account for the solvation energy, while temperature was controlled with the Andersen thermostat^54^. We ran a short DMD simulation (100,000 steps, i.e., ~5 ns) at the temperature of 0.35 kcal/mol kB (~250 K). During the simulation, all atomic coordinates were harmonically constrained, and only atoms in helices 5 and 6 at the surface of interactions of the heterodimer were allowed to move freely. We retrieved the lowest energy conformation as representative structure of the 6TM-MOR/β_2_-AR heterodimer.

### Calcium imaging

BE(2)-C cells were transiently transfected for 24 hours with pIRES-EGFP-EV alone or cotransfected with pIRES-EGFP-6TM-MOR/pIRES-EGFP-EV (6TM/EV), pIRES-EGFP-6TM-MOR/pOPRSVI/MCS-β_2_-AR (6TM/β_2_-AR), 7TM/pIRES-EGFP-EV (7TM/EV), pIRES-EGFP-7TM/pOPRSVI/MCS-β_2_-AR (7TM/β_2_-AR) or pOPRSVI/MCS-β_2_-AR/pIRES-EGFP-EV β_2_-AR/EV) expression plasmids. BE(2)-C cells or dissociated neurons were seeded onto glass coverslips, loaded with 1μM of the cell permeable calcium sensitive dye, Fura 2AM (Life Technologies) for 30 minutes and washed with PBS or HBSS before use. Coverslips containing cells were placed in a chamber with constant infusion of PBS or HBSS at room temperature as indicated below. Perfusion conditions for BE(2)-C cells were: 1 min PBS; 59 min 1, 3 or 10μM IBNtxA (SRI Int. Menlo Park, CA, USA) or 1 min PBS-3 min 10 μM naloxone or 1μM ICI 118,551 (Sigma Aldrich); -56 min 10 μM naloxone/1μM IBNtxA or 1μM ICI 118,551/1μM IBNtxA as indicated. Perfusion conditions for mouse trigeminal and dorsal root ganglia neurons were: 1 min HBSS, 18 min 50μM IBNtxA or 1 min HBSS-3 min 0.3μM Betaxolol HCl (Tocris Bioscience), or 0.3μM ICI 118,551 or 0.1μM SR-58611A (Tocris Bioscience). For neuronal imaging, 75mM KCl was used as a positive control at the end of the recording to ensure the responsiveness of the recorded cells. Fluorescence was detected by a Nikon Eclipse TI microscope at 340 and 380 excitation wavelengths and analyzed with the TI Element Software (Nikon). Cells were considered as responsive to a drug infusion if the 340/380 ratio was ≥0.2 from baseline. Mean values were compared using one-ANOVA, Sidak’s multiple comparison test or Student t test.

### *In vivo* studies

#### Animals

Naïve young adult (6–14 week old) outbred CD-1 (ICR: Crl) mice (Charles River) were used in drug interaction (isobolographic) studies. Approximately equal numbers of males and females were used in these behavioral experiments. Mice were housed in groups of 2 to 4, with same-sex littermates in standard polycarbonate cages. Subjects were housed in a temperature (20 °C ± 1 °C) and light-controlled (14:10 h light/dark cycle; lights on at 07:00 h) environment with tap water and food (Harlan Teklad 8604) available ad libitum. All procedures were consistent with appropriate national guidelines and approved by the McGill Downtown Animal Care and Use Committee.

For OIH studies C57bl/6J male mice (Harlan, Netherlands) were used. Mice were 7 weeks old upon the arrival and were allowed to recover for 7 days before the experiments. The mice were housed in plastic cages (n = 1–3/cage) with controlled ambient temperature of 23 ( ± 2)°C. Light-dark cycle was fixed to 12 h and all experiments were conducted during the light phase of the day, between 13:00 and 18:00 h. Water and standard laboratory chow were available ad libitum. Animals were not pre-selected for nociceptive experiments. After 5 days of habituation to the testing environment and equipment, sensitivity to mechanical stimuli was assessed. Based on baseline mechanical sensitivity, mice were allocated to the balanced treatment groups. Experiments were conducted in accordance with local and European regulation, guidelines of the International Association for the Study of Pain^58^ and 3R principles. Experiments were conceptually approved by the provincial government of Southern Finland (Etelä-Suomen aluehallintovirasto, Hämeenlinna, Finland, ESAVI/7929/04.10.07/2014).

#### Drugs

IBNtxA (0.25–5.0 mg/kg, SRI Int. Menlo Park, CA, USA), SR 59230A (5.0–100.0 mg/kg, Abcam), Betaxolol HCl (1.0–50.0 mg/kg, Abcam) and ICI 118,551 (10.0–100.0 mg/kg, Sigma Aldrich) were dissolved in physiological saline and administered intraperitoneally in a 10 ml/kg injection volume in the formalin model. Experimenters were blinded to drug and dose during testing. For OIH studies morphine hydrochloride was obtained from the University Pharmacy (Helsinki, Finland); morphine, IBNtxA and ICI 118,551 in this study were dissolved in physiological saline (0.9%) and administered in volume of 100 μl subcutaneously.

#### Formalin test for synergy between IBNtxA and β_1_-, β_2_-, and β_3_-AR antagonists

Mice were habituated in 8 individual Plexiglas cylinders placed on top of a glass surface suspended over high-resolution video cameras for at least 30 min prior to experimentation. Mice were then removed from their cylinder, injected with IBNtxA, betaxolol, ICI 118,551 or SR59230A alone, or in cocktails of equipotent doses (interpolated from the single drug dose-response curves) and placed back in the cylinder. Fifteen min later, all subjects were injected subcutaneously into the left plantar hind paw with 5% formalin (20 μL volume). Mice were subsequently videotaped for 60 min, and videotape observations were later sampled for 10 sec at 1-min intervals, with an observer blind to experimental condition recording the presence or absence of left hindpaw licking/biting in that 10-sec period. The early phase of the formalin test was defined as 0 to 10 min after injection, and the late phase as 10 to 6 min post-injection. Data are presented as the percentage of samples licking in either the early and late phase of the formalin test. In statistical analysis, a criterion α = 0.05 was adopted. Dose-response curves were created for each β_1_-, β_2_-, and β_3_-AR antagonists alone, and for IBNtxA. Half-maximal analgesic doses (AD_50_s) and associated 95% confidence intervals (CIs) and potency ratios were calculated using the method of Tallarida and Murray^59^, as implemented by the FlashCalc 40.1 macro (M. H. Ossipov, University of Arizona). We tested possible synergism between IBNtxA and the β_1_-, β_2_-, and β_3_-AR antagonists using isobolographic analysis. First, AD_50_ values we determined for each drug administered alone. Equipotent doses of IBNtxA and each β-adrenergic antagonist were then calculated based on the AD_50_ dose-ratio, yielding a ratio of 1:13 between both IBNtxA and betaxolol and IBNtxA and ICI 118, 551, and a 1:10 ratio between IBNtxA and SR59230A. IBNtxA/β antagonist cocktails were then mixed at these ratios and administered to mice as described above. To determine whether the interaction between IBNtxA and the β_1_-, β_2_-, and β_3_-AR antagonists is synergistic, we used the AD_50_s of each drug separately to determine a theoretical additive value for the combination, and compared this value to the experimental AD_50_ of the cocktail we recorded. If the observed AD_50_ was significantly lower (p < 0.05) than the theoretical additive value, and then the interaction was confirmed to be synergistic. Isobolographic analyses were performed using the method of Tallarida and Murray^59^, and calculated with the FlashCalc 40.1 macro (M. H. Ossipov, University of Arizona). GraphPad Prism 6.0 was used to create isobolograms as a visual representation of the interactions.

#### Nociceptive tests in OIH model

Schedule of opioid administration and morphine dose were selected based on the studies of Liang *et al.* 2006; 2007, as they have been reported to produce profound opioid induced hyperalgesia. Animals were administered morphine (20mg/kg) or IBNtxA (2mg/kg), or vehicle twice a day (8 am and 16 pm) for three consecutive days. For the fourth and fifth days the dose was increased to 40 mg/kg (morphine) and 4 mg/kg (IBNtxA). IBNtxA dose was selected based on the assessments of its acute analgetic potency in this study (formalin test, Fig. 4) and earlier studies^7^,^60^. Nociceptive sensitivity was assessed on the fifth day of experiment, prior to drug administration. On the sixth day of experiment, animals were administered ICI 118,551 (3.25mg/kg) and nociceptive behavior was re-assessed 30 min later on the hot plate and 90–150 min later on the cold plate.

To measure mechanical allodynia, the mice were placed on a metal mesh and covered with individual transparent plastic domes enabling the observation of the nociceptive behavior and stimulation of the paw with a calibrated von Frey monofilament (North Coast Medical, Inc., Morgan Hill, CA). Monofilament was slowly brought into contact with skin, perpendicularly to the surface. After skin contact, force just sufficient to bend the filament was applied for 3 s to standardize the time-course of stimulation. Determined foot withdrawal and brisk shaking or licking of the paw not associated with locomotor movement were accepted as a nociceptive response. Monofilaments ranging 0.008–4 g were applied in ascending order. Each filament was applied five times with 2-second interval between stimulations, and 5 min interval between filaments. The response rate for each monofilament was calculated: if the animal did not respond to any of five stimulations, the response rate for the given filament was 0%. Consequently, if all of the five stimulations induced nociceptive responses, response rate was 100%. Further, response rates of all tested filaments were counted together yielding the cumulative response rate, which was calculated for each animal. Further, the cumulative response rate was calculated for each animal as 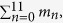 where *m*_*n*_ is an individual mass of n [0.008–4g]. To take into account individual mechanic sensitivity in baseline measurements, Δ cumulative response rate was calculated (Δ cum response rate = [the cumulative response rate after drug treatment]-[the cumulative response rate before drug treatment]). Δ cumulative response rate was used as an outcome measure in statistical analysis and group comparisons.

Thermal and cold sensitivity were assessed using hot/cold plate test (model: BIO-T2CT; Bioseb, Chaville, France). In the hot plate test, mice were placed on the heated metal surface and covered with transparent plexiglass dome. Licking or brisk shaking of the hind paw, or jumping, was considered as a sign of thermal nociception and latency to the first reaction was measured. To avoid tissue damage, the cut-off time was set to 60 s. Basal thermal nociception in the hot plate was assessed in morphine or IBNtxA treated animals on the fifth day of the experiment. The temperature of the hot plate was set to 50 °C (±0.2 °C), based on the results of pilot experiments. Cold nociception was assessed in similar manner at temperature of 3 °C or −5 °C for morphine or IBNtxA respectively, 30 min after the hot plate test. Temperatures were selected based on pilot experiments.

The level of significance was set at P value of 0.05. Prior to main analysis, the data gathered from nociceptive assays of OIH-experiments was checked and confirmed to meet the assumptions of parametric analysis. Normality of distribution was tested with Shapiro-Wilk normality test and the equality of variances with F-test. In assays of thermal and mechanical nociception, there were no differences between the results of vehicle groups of IBNtxA and morphine experiments. This allowed us to pool the results of vehicle groups in order to increase statistical power. In the comparisons between the treatment groups, mean values were compared using Student two-tailed *t* test and two-way ANOVA combined with Tukey’s multiple comparison test, depending on the number of groups. GraphPad Prism 6.0 was used for all data analyses.

## Additional Information

**How to cite this article**: Samoshkin, A. *et al.* Structural and functional interactions between six-transmembrane µ-opioid receptors and β_2_-adrenoreceptors modulate opioid signaling. *Sci. Rep.* **5**, 18198; doi: 10.1038/srep18198 (2015).

## Supplementary Material

Supplementary Information
