# Supplementary material for: Structural and functional interactions between six-transmembrane μ-opioid receptors and β2-adrenoreceptors modulate opioid signaling
Source: Sci Rep. 2015 Dec 11;5:18198. doi: 10.1038/srep18198 (PMC4676002; doi:10.1038/srep18198)
Supplement: Supplementary Information [file srep18198-s1.pdf]

Structural and functional interactions between six-transmembrane μ-opioid receptors and β_2-adrenoreceptors modulate opioid signaling

Authors

Alexander Samoshkin, Marino Convertino, Chi T. Viet, Jeffrey S. Wieskopf, Oleg Kambur, Jaclyn Marcovitz, Pinkal Patel, Laura S. Stone, Eija Kalso, Jeffrey S. Mogil, Brian L. Schmidt, William Maixner, Nikolay V. Dokholyan and Luda Diatchenko

Supplementary Information

Supplementary Table 1. Residues predicted to mediate the interaction between 6TM-MOR and β_2-AR in the formation of the 6TM-MOR/β_2-AR heterodimer.

6TM-MOR Mutant#	Helix 5	Helix 6	Helix 7
1[*]	I234A	-	-
2[*]	I234A, I256A	I298A, L305A	-
3[*]	L231A, I234A, I238A	-	-
4[*]	L231A, I234A, I238A, M243A, L257A	-	-
5[*]	I234A, I256A, L246A	I298A, L305A, V306A	-
6[*]	V126A, L116A, L121A, L129A	-	I322A

β_2-AR Mutant#	Helix 3	Helix 5	Helix 6
1[§*]	-	I201A	-
2[§]	-	I201A,V216A	I291A, V295A
3[*]		I205A, S220A, Q224A	L287A, I298A
4[**]	V117I	-	-
5[**]	-	-	F289Y
6[**]	-	-	F290T
7[**]	V117I	-	F289Y, F290T

[*]Alanine-mutated residues in β_2-AR and 6TM-MOR did not disrupt the 6TM-MOR/β_2-AR heterodimer; 6TM-MOR translocated to the cell surface when co-expressed with β_2-AR mutant (**S**upplementary Fig. S1). [§]Alanine-mutated residues in β_2-AR disrupted the 6TM-MOR/β_2-AR heterodimer; 6TM-MOR localized in the intracellular compartments when co-expressed with β_2-AR mutant (**S**upplementary Fig. S1). [§*]Alanine-mutated residue in β_2-AR reduced the 6TM-MOR/β_2-AR heterodimerezation. 6TM-MOR was not effectively translocated to the cell surface when co-expressed with β_2-AR mutant. [**]Mutated residues in β_2-AR did not disrupt the 6TM-MOR/β_2-AR heterodimer. These predicted neutral mutations were used as a negative control because they are located on the surfaces that do not mediate the 6TM-MOR/β_2-AR interaction.

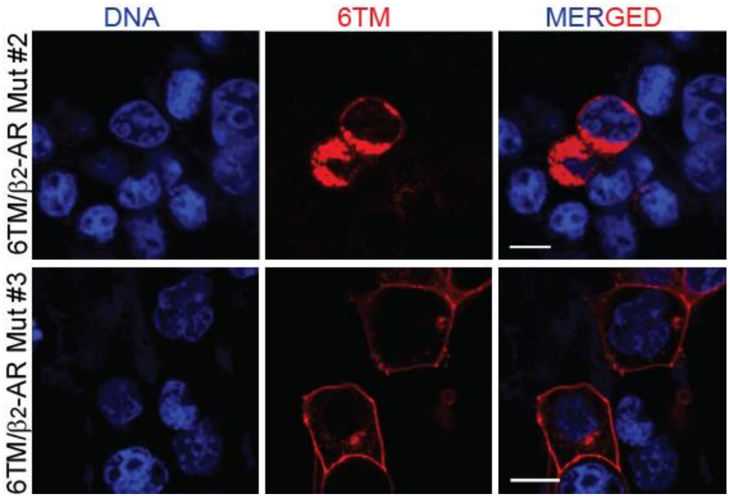

Supplementary Figure S1. Confocal images of HEK293 cells co-expressing FLAG-tagged 6TM-MOR with the β_2-AR mutants 2) I201A, V216A, I291A, V295A (Mut#2) and 3) O205A, S220A, Q224A, I298A, L297A (Mut#3). 6TM-MOR (red) retained inside the cells upon co-transfection with the β_2-AR Mut#2 (upper row) or but translocated to the cell surface with the β_2-AR Mut#3 (lower row). Scale bar 10µm.

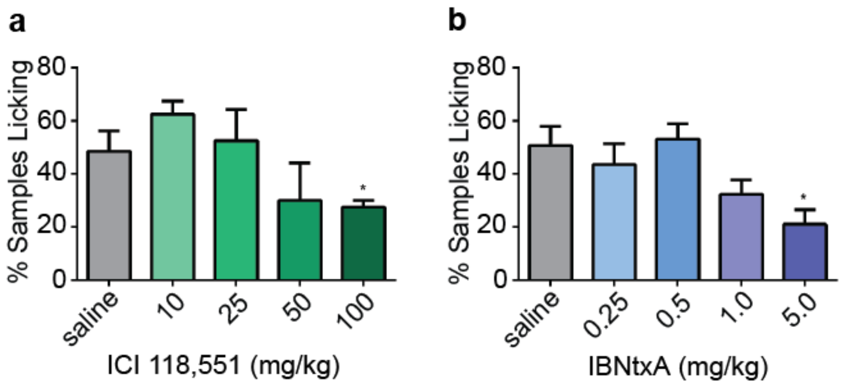

Supplementary Figure S2. Analgesic effects of ICI 118,551 and IBNtxA. **(a)** β_2-AR antagonist ICI 118,551 and **(b)** 6TM-MOR ligand IBNtxA produce dose-dependent analgesia in the early phase (0-10 min post-injection) nocifensive behaviors after 5% formalin injection into the plantar hind paw. One hundred mg/kg doses of ICI 118,551 and 5 mg/kg doses of IBNtxA significantly inhibit pain behavior compared to saline. Error bars represent SEM. *$p<0.05$ differ from saline.

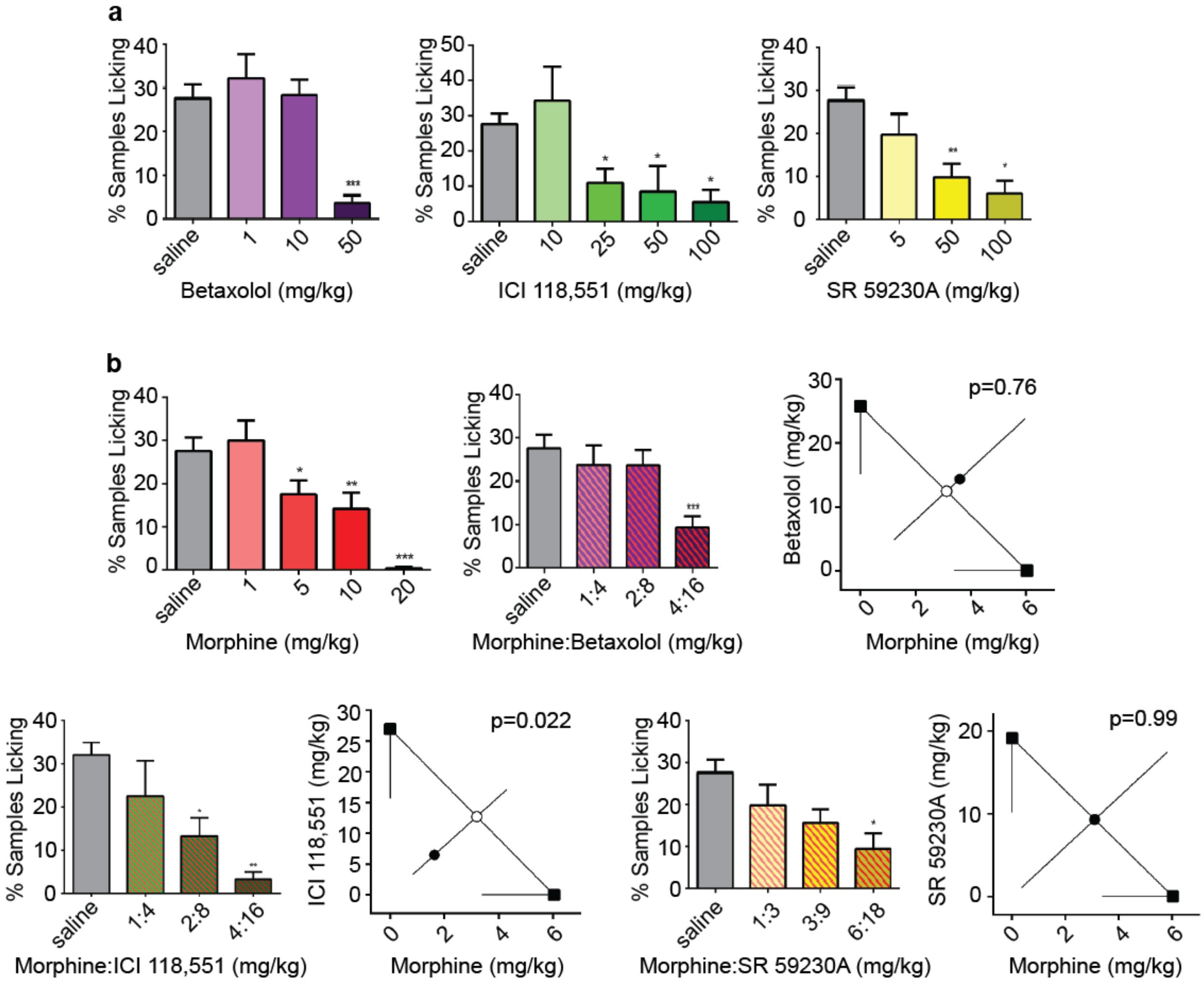

Supplementary Figure S3. Analgesic effects upon administration of β-antagonists alone or with morphine co-administration. (a) β-AR antagonists: betaxolol (β_1-AR), ICI 118,551 (β_2-AR) and SR 59230A (β_3-AR) produce dose-dependent analgesia in the late phase (10-60 min post-injection) nocifensive behaviors after 5% formalin injection into the plantar hind paw; bars represent mean percentage (+ SEM). **(b)** Co-administration of ICI 118,551 and morphine produces synergistic analgesia. The bar graph displays the dose response of the cocktail tested, and the isobolographic analysis tests the synergistic relationship of morphine and selective β-antagonists. Neither β_1-AR antagonist betaxolol nor β_3-AR antagonist SR 59230A show a synergistic interaction with morphine (p=0.76 and 0.99, respectively), while isobolographic

analysis of morphine and ICI 118,551 shows the synergistic relationship of these two drugs ($p=0.022$). *$p<0.05$, **$p<0.01$, ***$p<0.001$ differ from saline.

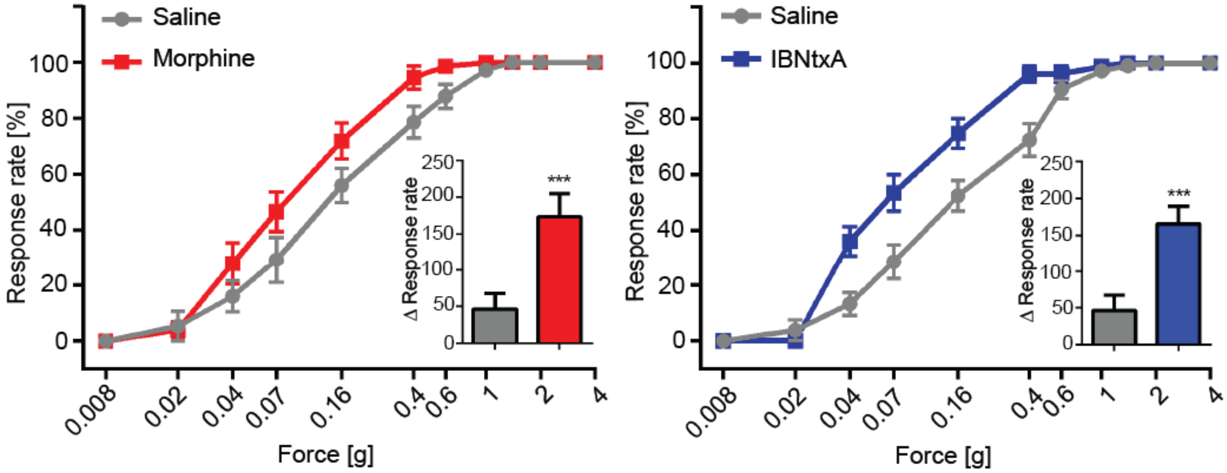

Supplementary Figure S4. Chronic morphine or IBNtxA administration produces OIH assessed in the mechanical test. C57BL/6J mice received 4 days of morphine (20 mg/kg days 1-3, 40mg/kg day 4, s.c., twice a day, n=15), or IBNtxA (2 mg/kg days1-3, 4 mg/kg day 4, s.c., twice a day, n=15) treatment to induce OIH or vehicle (saline, s.c., n=12). Panel represents increased response rates after mechanical stimulation in von Frey test as a sign of OIH (morphine, left panel; IBNtxA, right panel); inserted graphs show differences in cumulative response rate. Error bars represent SEM. ***p<0.001 vs. saline (Student two-tailed *t* test).
